# Spatial metabolomic mapping reveals peel-color- and tissue-region-associated metabolic heterogeneity in postharvest ‘Yanzhihong’ apricot fruit

**DOI:** 10.3389/fpls.2026.1906148

**Published:** 2026-08-20

**Authors:** Xueying Shi, Yang Zhao, Wenbao Jia, Haidong Zhang, Aoqi Cui, Yanhong He

**Affiliations:** 1 College of Forestry, Inner Mongolia Agricultural University, Hohhot, China; 2 College of Forestry, Inner Mongolia Agricultural University, Hohhot, Inner Mongolia, China

**Keywords:** mass spectrometry imaging, metabolic heterogeneity, postharvest apricot fruit, red and yellow fruit surfaces, spatial metabolomics, ‘Yanzhihong’ apricot

## Abstract

**Introduction:**

Spatial metabolic heterogeneity associated with fruit color and tissue type remains insufficiently characterized in postharvest apricot fruit. In this study, MALDI-MSI-based spatial metabolomics was used to characterize color- and tissue-associated ion-response heterogeneity in postharvest ‘Yanzhihong’ apricot fruit.

**Methods:**

RMS-normalized ion responses were analyzed using spatial shrunken-centroid segmentation, principal component analysis (PCA), exploratory orthogonal partial least-squares discriminant analysis (OPLS-DA), fold-change analysis, and sample-level Pearson correlation assessment.

**Results:**

Clear overall differentiation was observed between the red- and yellow-colored sides, indicating color-associated spatial metabolic heterogeneity within the fruit. Exploratory differential-feature screening and subsequent spatial analyses highlighted candidate ion features related to monolignol/phenylpropanoid metabolism, phenolic acids, flavonols, sugar phosphates, nucleotide sugars, and membrane lipids. Candidate ion features tentatively assigned to caffeyl alcohol, 4-hydroxycinnamic acid, and dihydroferulic acid, together with most selected membrane-lipid-related candidate features, showed stronger relative responses on the yellow-colored side. In contrast, candidate ion features tentatively assigned to myricetin-3-O-(4″-acetyl)rhamnoside and UDP-GalNAcA showed stronger relative responses on the red-colored side. Among the selected monolignol/phenylpropanoid-, phenolic-acid-, and flavonol-related candidate ion features, the 4-hydroxycinnamic-acid-related ion was the only feature that showed the same regional ordering on both fruit sides, with lower mean responses in the flesh than in the peel and near-stone regions. Other regional mean differences were feature dependent and were not statistically validated at the current sample size.

**Discussion:**

These findings characterize candidate color- and tissue-associated ion-response features under the sampled postharvest condition. Given the single cultivar, three paired biological replicates, and one postharvest sampling point, the findings remain exploratory and do not establish postharvest-quality markers.

## 1 Introduction

Apricot (*Prunus armeniaca* L.) is an economically important stone fruit whose metabolic composition continues to change after harvest. Metabolomic studies of postharvest fruit have shown that primary and secondary metabolites are associated with ripening, senescence, and responses to storage conditions (Yun et al., 2022). However, conventional metabolomic analyses generally rely on homogenized tissues and consequently provide limited information on the spatial distribution of metabolites within individual fruits. This limitation is particularly relevant for fruits with heterogeneous surface coloration and anatomically distinct tissue regions, in which bulk measurements may obscure localized metabolic variation.

Mass spectrometry imaging (MSI) enables molecular distributions to be examined directly in tissue while retaining spatial information. The feasibility of MALDI-based molecular imaging in tissue was established in early methodological work (Caprioli et al., 1997), and the approach has subsequently been extended to plant tissues, where sample preparation, matrix selection, ion suppression, and metabolite identification remain important analytical considerations (Qin et al., 2018; Chen et al., 2024; Vats et al., 2024; Yin et al., 2024). Applications in fruit tissues have demonstrated species- and tissue-dependent spatial metabolic patterns. MALDI-MSI has been used to visualize soluble carbohydrate distributions in apple fruit (Horikawa et al., 2019), developmental-stage-associated spatial distributions of endogenous molecules in wolfberry fruit (Zhao et al., 2021), maturity-related changes in sugars, organic acids, and phenolic-related metabolites in jujube fruit (Lu et al., 2023), and cultivar- and tissue-region-associated distributions of carbohydrates and organic acids in strawberry fruit (Fujiki et al., 2025). These studies show that MSI can resolve localized metabolic variation that may be obscured by homogenized-tissue analysis. Nevertheless, ionization efficiency, matrix effects, and ion suppression vary among compounds, and metabolite annotation remains an important source of uncertainty. Accordingly, MSI signals are more appropriately interpreted as feature-specific relative ion-response patterns unless compound identity and abundance are independently confirmed.

Phenylpropanoid metabolism provides precursors for a range of hydroxycinnamic acids, monolignols, flavonoids, and other aromatic metabolites, with pathway output varying among organs, developmental stages, and plant species (Vogt, 2010); recent work has further characterized transporter-mediated caffeyl alcohol uptake during C-lignin biosynthesis (Zhuo et al., 2025). Flavonoid biosynthesis represents a major branch of this network and contributes to chemically diverse flavones, flavonols, anthocyanins, and related compounds (Winkel-Shirley, 2001). Cross-species studies further show that flavonol composition can vary substantially among fruit and nut tissues; for example, cultivar-dependent differences in flavonol glycosides have been reported in almond seedcoats (Frison-Norrie and Sporns, 2002). These observations provide a broader biochemical context for examining individual phenylpropanoid- and flavonol-related candidate ion features in apricot fruit.

Recent metabolomic and transcriptomic studies have demonstrated that flavonoid and flavonol composition in apricot fruit varies with genotype and developmental stage (Chen et al., 2023; Han et al., 2023). More importantly, a recent MALDI-IMS study of the same ‘Yanzhihong’ cultivar demonstrated that the visually distinct red- and yellow-colored regions of individual fruits also differed in the spatial distributions of sugars, organic acids, carotenoids, and anthocyanin-related metabolites (Zhao et al., 2025). These findings indicate that the contrasting surface coloration of ‘Yanzhihong’ apricot is accompanied by measurable biochemical heterogeneity and therefore provides a biologically grounded within-fruit contrast for spatial metabolomic investigation. However, whether this color-associated heterogeneity extends to monolignol/phenylpropanoid-related, phenolic-acid-related, flavonol-related, sugar-phosphate-related, nucleotide-sugar-related, and membrane-lipid-related ion features, and whether individual features show additional tissue-region-associated variation, remains insufficiently characterized.

Accordingly, the present study used MALDI-MSI to characterize color- and tissue-associated variation in normalized ion-response profiles of ‘Yanzhihong’ apricot fruit at a single postharvest sampling point. Candidate ion features from these chemical categories were examined between paired red- and yellow-colored sides and among anatomically defined peel, flesh, and near-stone regions.

## 2 Materials and methods

### 2.1 Experimental materials

‘Yanzhihong’ apricot fruits were collected in July 2024 from the Wanjiagou Demonstration Orchard in Hohhot, Inner Mongolia, China. Three fruits with comparable developmental and maturity status, similar size, intact appearance, and no visible disease, insect injury, or mechanical damage were selected as independent biological replicates. Each selected fruit exhibited a clearly distinguishable red-colored side and yellow-colored side. The appearance of all three biological replicate fruits and the corresponding paired red- and yellow-colored sampling positions are shown in **Supplementary Figure 1**.

Tissues from the red- and yellow-colored sides of each fruit were collected as paired samples and designated red_1–yellow_1, red_2–yellow_2, and red_3–yellow_3. The two colored regions were processed separately and immediately snap-frozen in liquid nitrogen. Samples were transported to the laboratory on dry ice within approximately 0.5 h and stored at −80 °C until cryosectioning and MALDI-MSI analysis.

### 2.2 Tissue cryosectioning and sample preparation for mass spectrometry imaging

Apricot tissue blocks were equilibrated at −20 °C for 1 h before cryosectioning. Fresh apricot mesocarp is highly hydrated, soft, and mechanically fragile, and thinner sections were difficult to obtain and transfer without cracking, curling, or loss of tissue continuity. A section thickness of 50 μm was therefore selected as a practical tissue-preparation condition to preserve section integrity during transfer, matrix application, and MALDI-MSI acquisition. Plant tissues differ substantially from mammalian tissues in their sectioning properties. In particular, water-rich plant tissues are prone to crumbling and fracturing during preparation, and section thicknesses around 50 μm have been commonly used in plant MSI studies as a compromise between tissue integrity and analytical practicality (Dong et al., 2016). A 50 μm cryosection thickness has also been used for MALDI-MSI of water-rich pineapple fruit tissue (Suarez et al., 2023). More recent plant MSI workflows similarly include cryosection thicknesses in the range of approximately 30–50 μm (Horn and Chapman, 2024).

No thickness-gradient experiment using thinner sections of the same apricot tissue was performed in the present study. Therefore, the selected 50 μm thickness should not be interpreted as experimentally demonstrated to be superior to thinner sections or as a universally optimal MALDI-MSI condition. Rather, it represents a tissue-specific preparation condition selected to maintain the structural integrity of the present apricot samples. Tissue-section thickness can influence MALDI-MS signal generation and imaging performance in a tissue- and instrument-dependent manner (Wang et al., 2023). The suitability of the sections used in the present study was assessed from preservation of tissue morphology, a well-defined mean mass spectrum, and interpretable unsmoothed spatial ion-distribution patterns (**Supplementary Figure 2**).

### 2.3 Matrix spraying

A 15 mg/mL DHB (2, 5-dihydroxybenzoic acid) solution was prepared using acetonitrile/water (90:10, v/v) as the solvent. The DHB matrix solution was uniformly sprayed onto the ITO slides containing tissue sections using a TM-Sprayer matrix sprayer. The instrument parameters were as follows: temperature, 60 °C; flow rate, 0.1 mL/min; gas pressure, 7 psi; and 25 spraying cycles in total. The drying time between cycles was 5 s.

### 2.4 Mass spectrometry imaging detection and analysis

The matrix-coated ITO slides were analyzed using a Bruker timsTOF fleX mass spectrometry system (Bruker Daltonics, Bremen, Germany) equipped with a 10 kHz smartbeam 3D laser. All tissue sections were analyzed exclusively in positive ion mode. Laser power was set to 80% and maintained constant throughout the experiment. Tissue regions were selected using DataImaging software (Bruker), and the imaging raster step was set to 50 μm, corresponding to a nominal lateral pixel size of 50 μm × 50 μm. The mass acquisition range was set to m/z 50–1300, and each pixel spectrum was acquired using 400 laser shots. The same ionization mode, laser power, raster size, matrix-application conditions, and acquisition parameters were applied to all red- and yellow-colored samples to ensure analytical comparability between the two fruit surfaces.

The laser beam was directed onto the selected tissue regions to sequentially scan the predefined imaging pixels. Under laser irradiation, analytes were desorbed and ionized together with the matrix, and the resulting ions were detected by mass spectrometry to obtain the m/z values and corresponding signal intensities at each spatial coordinate. The raw mass-spectrometric data were subsequently imported into SCiLS Lab software and normalized using the root mean square (RMS) method (Deininger et al., 2011). This normalization generated relative ion-intensity information for individual m/z features at each spatial point, which was subsequently visualized as spatial ion-distribution maps.

Raw spectra were RMS-normalized, and the resulting values were treated as relative ion responses rather than absolute metabolite concentrations. Because ionization efficiency and adduct formation vary among compounds, comparisons were restricted to the same ion feature measured under identical conditions on the red- and yellow-colored sides. Signal intensities were not directly compared across different metabolites or chemical classes. A weak or undetectable signal in positive-ion mode was not interpreted as evidence for the absence of the corresponding metabolite (Zemaitis et al., 2023).

For methodological context, the shrunken-centroid concept was originally introduced by Tibshirani et al. (2003), while spatially informed segmentation approaches were subsequently developed for mass spectrometry imaging data (Bemis et al., 2016, 2023). Spatially aware shrunken-centroid clustering was performed using r = 2, s = 0, and k = 10 as initial exploratory segmentation settings. The neighborhood radius r = 2 introduces short-range spatial information while limiting excessive smoothing across adjacent structures; s = 0 applies no additional centroid shrinkage; and k = 10 specifies the maximum number of candidate segments considered by the algorithm rather than the final number of anatomical regions. These parameters were not independently optimized against apricot histology and were therefore not used to define anatomical ROIs or as evidence of analytical quality. Instead, the peel, flesh, and near-stone ROIs used for subsequent comparisons were delineated independently according to visible tissue anatomy. Spatial segmentation was used solely to visualize spatially organized ion-response heterogeneity (Bemis et al., 2016, 2023).

### 2.5 Assessment of dataset consistency and signal stability

Dataset consistency was assessed independently of the spatial segmentation analysis. Pairwise Pearson correlation coefficients were calculated from the RMS-normalized whole-section ion-response profiles of all 1, 038 annotated ion features within each fruit-color group. Feature-level variability was additionally summarized using coefficients of variation (CVs). Five endogenous ion features consistently detected across all six datasets were selected as internal stability references, and their observed m/z, assigned molecular formula and adduct, theoretical m/z, mass error, and across-sample CV are reported in **Supplementary Table 1**. Because the original acquisition did not include a dedicated pooled-QC tissue section or external reference standard, these retrospective consistency and stability metrics were used to characterize dataset consistency and signal stability rather than being presented as a substitute for a dedicated experimental QC design (Huang et al., 2025). Because these metrics were calculated across biological samples, they reflect combined biological and analytical variability and should not be interpreted as estimates of technical reproducibility alone.

### 2.6 Metabolite annotation and confidence reporting

Metabolite annotation followed the workflow provided by the analytical platform. For ion features with on-tissue MS/MS spectra, precursor and fragment-ion information was matched against the platform-integrated MALDI, local, public, and, where applicable, in silico spectral resources. For features without MS/MS spectra, precursor m/z values were searched against in-house and public databases. According to the analytical-platform workflow, nominal precursor-matching tolerances were <500 ppm for MS/MS-supported matching and 10 ppm for MS1-based matching, with nominal matching-score criteria of >0.5 and >0.8, respectively.

Annotations supported by on-tissue MS/MS evidence were reported as MS/MS-supported candidate annotations, whereas those based on precursor-mass matching were reported as MS1-only candidate annotations. All assignments were treated as candidate annotations unless confirmed using authentic standards. Feature-specific adducts and platform-reported annotation information were retained in **Supplementary Table 2**. The original platform workflow did not provide a numerical S/N cutoff and did not use METASPACE. In total, 1, 038 candidate ion-feature annotations were reported, including 375 with on-tissue MS/MS support (Baquer et al., 2023; Eisenberg et al., 2023).

### 2.7 Data processing and statistical analysis

Unsupervised principal component analysis (PCA) was used to evaluate overall variation between the red- and yellow-colored samples. Orthogonal partial least-squares discriminant analysis (OPLS-DA) was performed in R using MetaboAnalystR v1.0.1 on log_2_-transformed and mean-centered ion-response data. Model characteristics were summarized using R²X, R²Y, and Q², and model stability was assessed using 200 random class-label permutations. VIP values derived from OPLS-DA were used only for exploratory candidate-feature prioritization and were interpreted together with PCA and fold-change patterns. According to the analytical-platform workflow, candidate differential ion features were initially screened using VIP > 1 and a two-sided univariate P < 0.05. Given the limited sample size, these criteria were used for exploratory feature screening rather than biomarker validation.

For the red-versus-yellow comparison, fold change was calculated from the group means of RMS-normalized ion responses as Log_2_FC = log_2_(mean response in the red-colored group/mean response in the yellow-colored group). Positive Log_2_FC values indicate higher relative responses on the red-colored side, whereas negative values indicate higher responses on the yellow-colored side.

**Figure 1:**
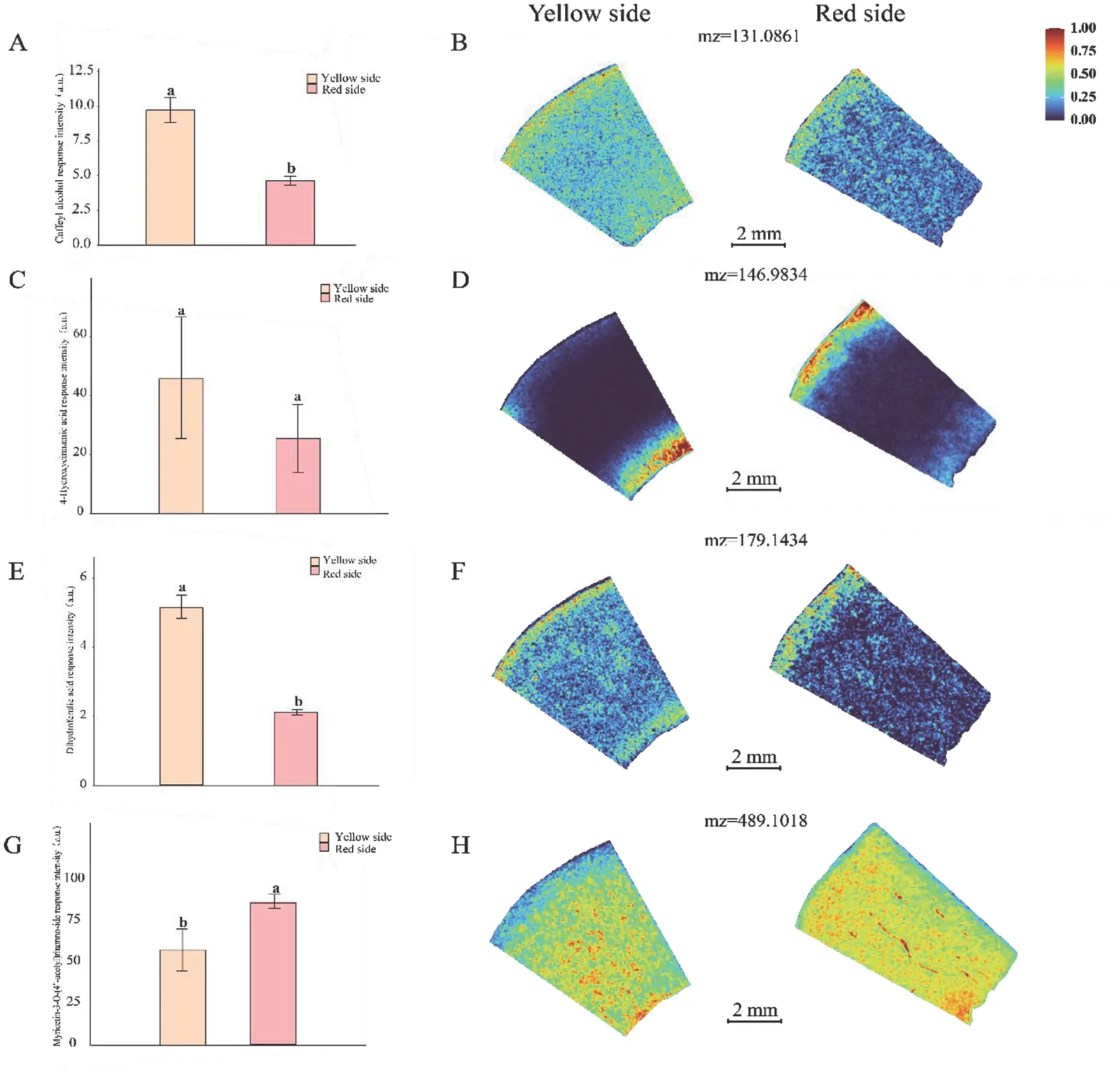
Relative responses and *in situ* spatial distributions of selected monolignol/phenylpropanoid-, phenolic-acid-, and flavonol-related candidate ion features on the red- and yellow-colored sides of apricot fruit. **(A, C, E, G)** show the comparisons of RMS-normalized relative responses of candidate ions tentatively assigned to caffeyl alcohol, 4-hydroxycinnamic acid, dihydroferulic acid, and myricetin-3-O-(4’’-acetyl)rhamnoside, respectively, between the yellow-colored and red-colored sides of apricot fruit. **(B, D, F, H)** show the corresponding ion-distribution maps at m/z 131.0861, 146.9834, 179.1434, and 489.1018 for the candidate ions tentatively assigned to caffeyl alcohol, 4-hydroxycinnamic acid, dihydroferulic acid, and myricetin-3-O-(4’’-acetyl)rhamnoside, respectively. Bar-chart data are presented as mean ± standard error from three paired biological replicates (n = 3 pairs). Differences between the yellow- and red-colored sides were assessed using two-sided paired Student’s t-tests. Different lowercase letters indicate a statistically significant difference at P < 0.05. In the mass spectrometry imaging maps, colors from blue to red indicate normalized relative signal intensity from low to high. Scale bars in panels **(B, D, F, H)** represent 2 mm.

**Figure 2:**
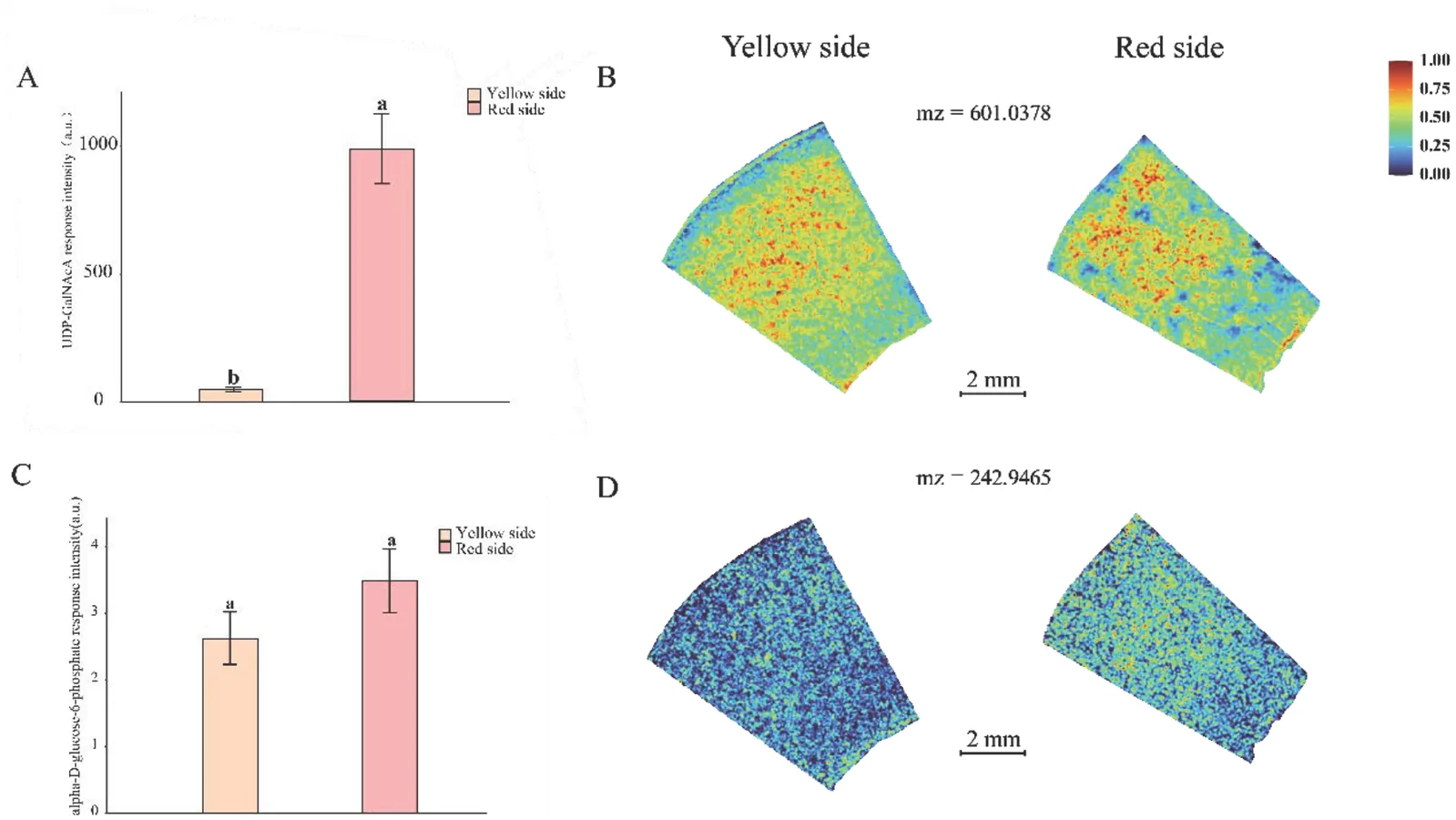
Relative responses and *in situ* spatial distributions of candidate sugar-phosphate- and nucleotide-sugar/glycosylation-related ion features on the red- and yellow-colored sides of apricot fruit. **(A, C)** show the RMS-normalized relative responses of the candidate UDP-GalNAcA-related and G-6-P-related ions, respectively. **(B, D)** show the corresponding ion-distribution maps at m/z 601.0378 and 242.9465, respectively. Bar-chart data are presented as mean ± standard error from three paired biological replicates (n = 3 pairs). For each ion feature, differences between the yellow- and red-colored sides were assessed using a two-sided paired Student’s t-test. Different lowercase letters within the same panel indicate a statistically significant difference at P < 0.05, whereas identical letters indicate no significant difference. Colors from blue to red indicate normalized relative ion responses from low to high. Scale bars in panels **(B, D)** represent 2 mm.

**Figure 3:**
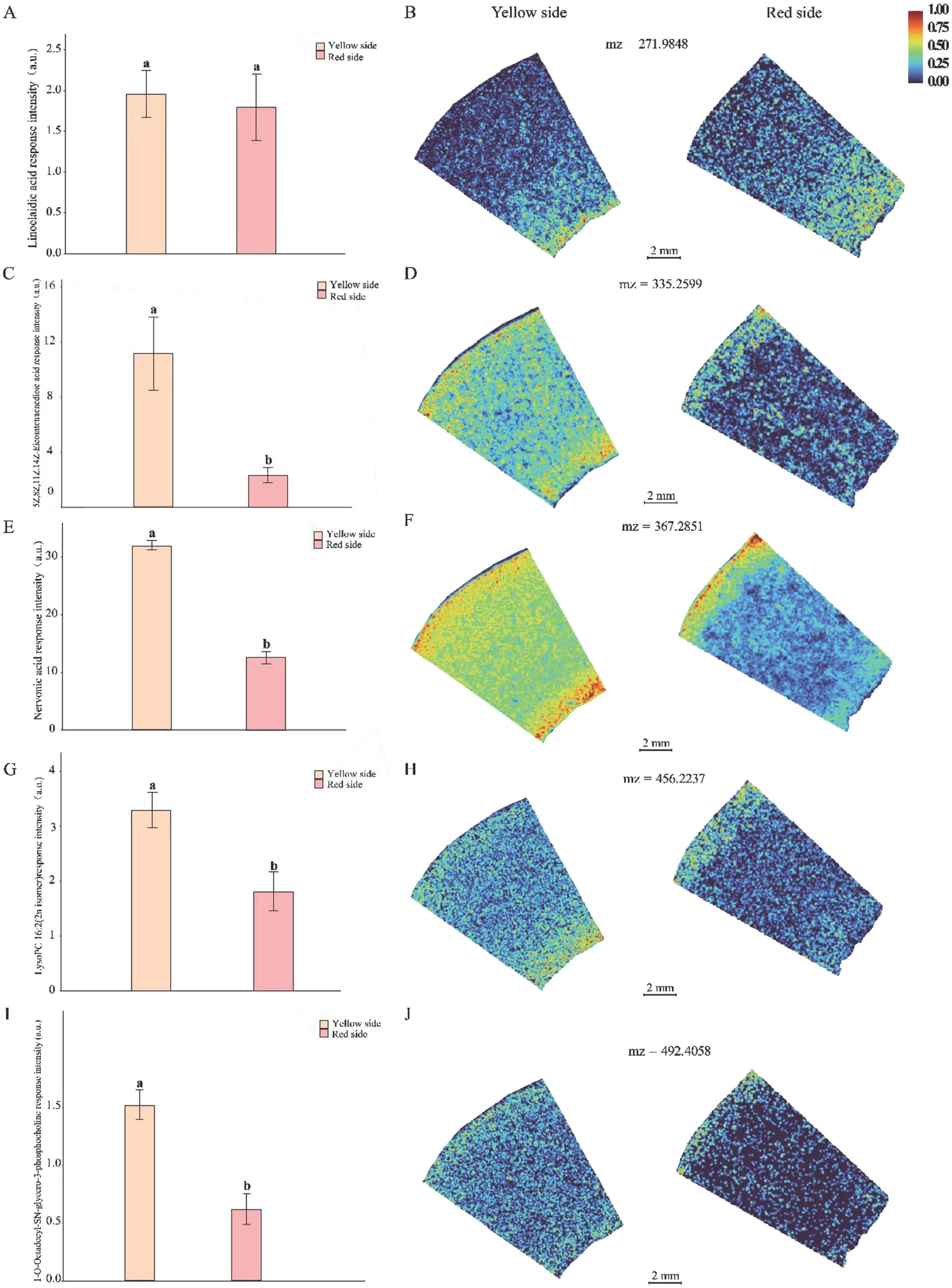
Relative responses and *in situ* spatial distributions of selected membrane-lipid-related candidate ion features and an unassigned ion feature on the red- and yellow-colored sides of apricot fruit. **(A, C, E, G, I)** show the RMS-normalized relative responses of the unassigned ion feature at m/z 271.9848 and candidate ions tentatively assigned to 5Z, 8Z, 11Z, 14Z-eicosatetraenedioic acid, nervonic acid, LysoPC 16:2(2n isomer), and 1-O-octadecyl-sn-glycero-3-phosphocholine, respectively. **(B, D, F, H, J)** show the corresponding ion-distribution maps. Bar-chart data are presented as mean ± standard error from three paired biological replicates (n = 3 pairs). For each ion feature, differences between the yellow- and red-colored sides were assessed separately using a two-sided paired Student’s t-test. Different lowercase letters within the same panel indicate a statistically significant difference at P < 0.05. Scale bars in panels **(B, D, F, H, J)** represent 2 mm.

Because the red- and yellow-colored samples were obtained from opposite sides of the same individual fruits, comparisons of the selected ion features shown in **Figures 1**–**3** were performed using a paired design. For each ion feature, the three corresponding red–yellow fruit pairs (red_1–yellow_1, red_2–yellow_2, and red_3–yellow_3) were compared using a two-sided paired Student’s t-test (n = 3 pairs). P < 0.05 was considered statistically significant. Given the limited number of biological replicates, these comparisons were interpreted as exploratory. The exact P values for the paired comparisons of the selected ion features shown in **Figures 1**–**3** are provided in **Supplementary Table 5**.

Pairwise Pearson correlation coefficients among the 50 platform-defined differential ion features with the largest absolute Log_2_FC values were calculated across the six whole-section samples. The underlying correlation coefficients, raw P values, and Benjamini–Hochberg FDR-adjusted q values are provided in **Supplementary Table 4** for reference. Because only three biological replicates were available per fruit side, comparisons among the peel, flesh, and near-stone ROIs were interpreted descriptively on the basis of mean ± standard error patterns. No class-wide or statistically validated regional effect was inferred solely from these n = 3 ROI comparisons.

## 3 Results

### 3.1 Dataset consistency and global metabolic variation

Before evaluating color- and tissue-associated metabolic patterns, the consistency of the MALDI-MSI dataset was assessed using the RMS-normalized whole-section ion-response profiles. Pairwise Pearson correlation coefficients ranged from 0.9483 to 0.9906 among the three yellow-colored-side replicates and from 0.9885 to 0.9959 among the three red-colored-side replicates, indicating good within-group consistency.

Feature-level analysis showed median CV values of 12.83% and 13.98% across the 1, 038 annotated ion features in the yellow- and red-colored groups, respectively. In addition, 74.47% of the ion features in the yellow-colored group and 77.65% in the red-colored group showed CV values below 20%. The five endogenous reference ion features showed across-sample CV values of 3.06%–10.83%, with absolute mass errors ranging from 0.05 to 3.02 ppm (**Supplementary Table 1**). These results indicate good overall consistency of the normalized ion-response profiles across the biological replicates.

**Figure 4:**
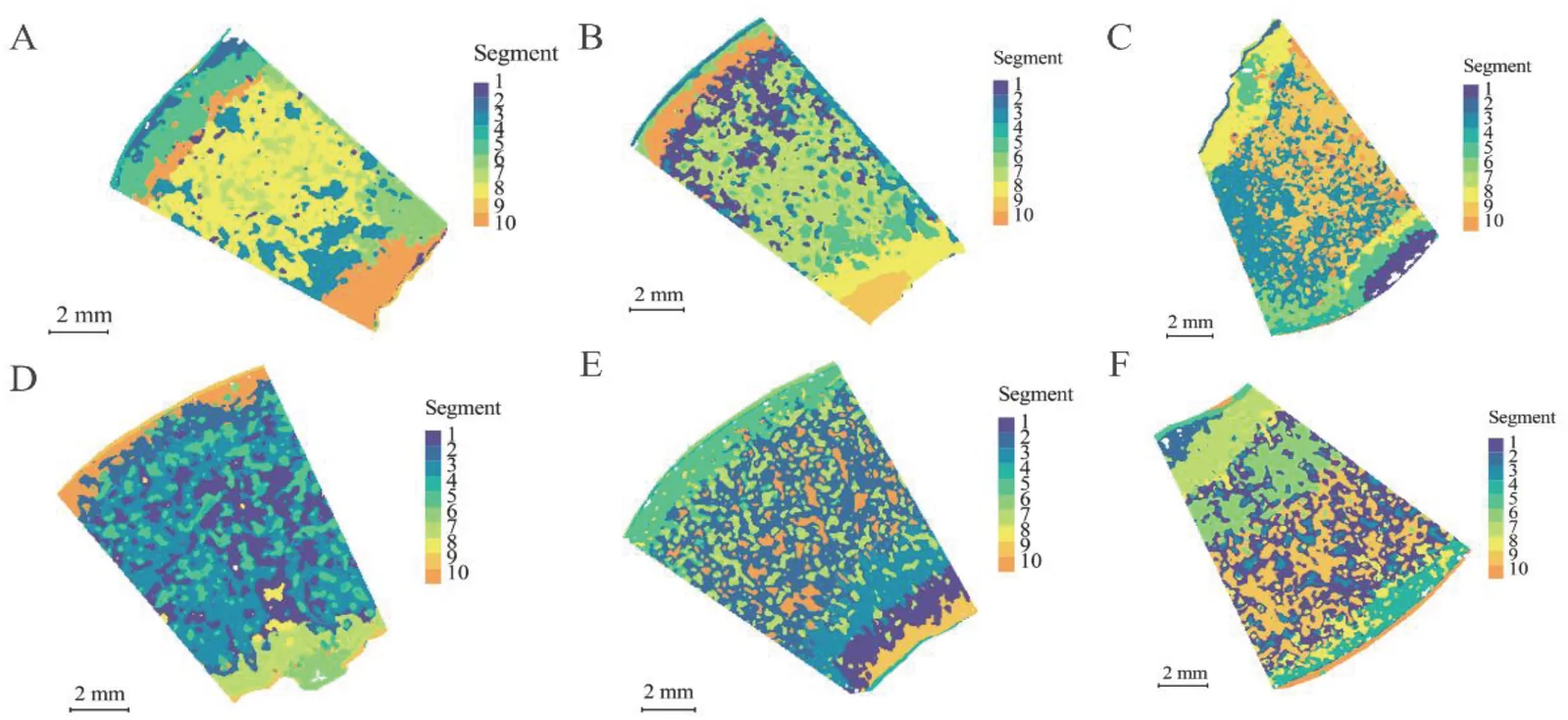
Spatial shrunken-centroid segmentation maps of all biological replicates of ‘Yanzhihong’ apricot fruit based on MALDI-MSI data. **(A–C)** Spatial segmentation maps of red_1, red_2, and red_3, respectively. **(D–F)** Spatial segmentation maps of yellow_1, yellow_2, and yellow_3, respectively. Samples arranged in the same column were obtained from the red- and yellow-colored sides of the same individual fruit, corresponding to three paired biological replicates. Spatial shrunken-centroid clustering was performed using r = 2, s = 0, and k = 10. The segmentation maps were used for exploratory visualization only. Anatomical ROIs were defined independently according to visible tissue structure.

Spatial shrunken-centroid segmentation further revealed structured variation in ion-response patterns across the tissue sections (**Figure 4**). Because anatomical ROIs were defined independently from visible tissue structure, the segmentation maps were used to visualize spatial metabolic organization rather than to define the peel, flesh, and near-stone regions.

**Figure 5:**
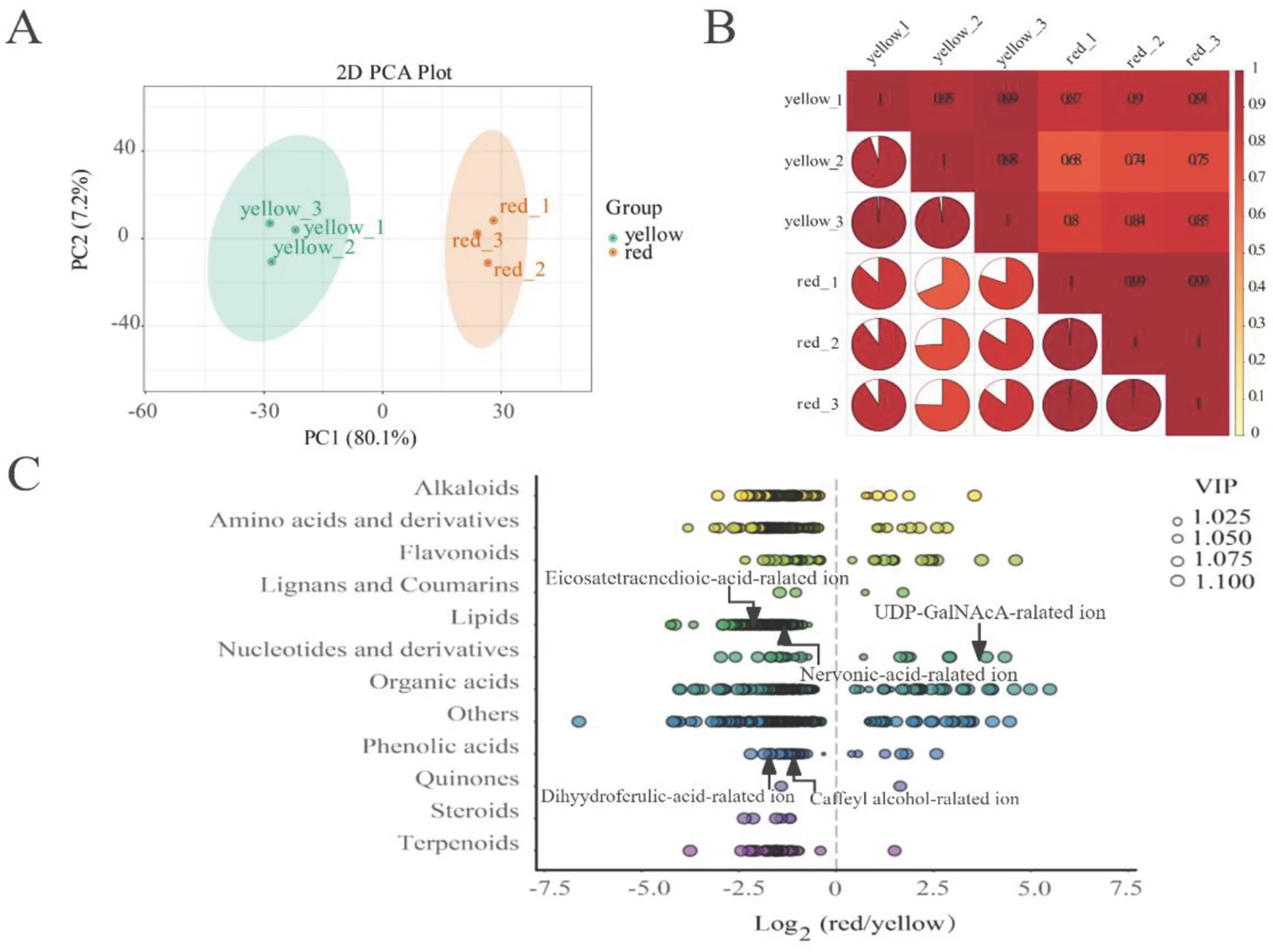
Dataset-level multivariate analysis and exploratory differential-feature screening between the red- and yellow-colored sides of apricot fruit. **(A)**, PCA score plot based on the six whole-section samples, including three biological replicates from the yellow-colored side (yellow_1–yellow_3) and three paired replicates from the red-colored side (red_1–red_3). PC1 and PC2 explained 80.1% and 7.2% of the total variance, respectively. **(B)**, pairwise sample correlation matrix showing the similarity among the six whole-section samples. Numerical values represent pairwise correlation coefficients, and the corresponding circular sectors provide a graphical representation of correlation strength. **(C)**, Log_2_FC–VIP plot of platform-defined differential ion features. Differential ion features were screened according to the analytical-platform criteria of VIP > 1 and P < 0.05. Log_2_FC was calculated as log_2_(mean RMS-normalized response on the red-colored side/mean RMS-normalized response on the yellow-colored side); positive values indicate higher responses on the red-colored side, whereas negative values indicate higher responses on the yellow-colored side. Chemical classes are shown on the y-axis, and point size represents the VIP score. Selected representative candidate ion features discussed in the text are labeled. Complete feature-level information is provided in **Supplementary Table 3**.

PCA showed clear overall differentiation between the red- and yellow-colored sides of the fruit (**Figure 5A**). PC1 and PC2 explained 80.1% and 7.2% of the total variation, respectively. The sample correlation matrix showed high within-group similarity and lower similarity between the two color groups (**Figure 5B**), supporting the presence of color-associated differences in the overall normalized ion-response profiles.

### 3.2 Exploratory screening of candidate differential ion features between the red- and yellow-colored sides

OPLS-DA was used as an exploratory supervised analysis of group-associated variation. The model yielded R²X = 0.871, R²Y = 1.000, and Q² = 0.992. In the 200-permutation assessment, the permutation-derived P values were 0.02 for Q² and 0.545 for R²Y; four permuted models showed Q² values exceeding that of the original model, whereas 109 showed R²Y values exceeding that of the original model. Given the small sample size and the non-significant permutation result for R²Y, the OPLS-DA model was not treated as stand-alone evidence of robust class discrimination. Instead, VIP scores were used only for exploratory candidate prioritization in conjunction with PCA and fold-change patterns. Among the platform-defined differential ion features, candidate ions tentatively assigned to caffeyl alcohol and dihydroferulic acid showed higher relative responses on the yellow-colored side (Log_2_FC < 0). Other selected candidate ions examined in the subsequent spatial analyses, including those tentatively assigned to 4-hydroxycinnamic acid and myricetin-3-O-(4’’-acetyl)rhamnoside, were retained as biologically relevant candidate features but did not meet the joint platform-defined screening criterion of VIP > 1 and P < 0.05. **Figure 5C** summarizes the platform-defined differential features according to chemical class, Log_2_FC, and VIP.

### 3.3 Side- and tissue-associated response patterns of selected monolignol/phenylpropanoid-, phenolic-acid-, and flavonol-related candidate ion features

Selected monolignol/phenylpropanoid-, phenolic-acid-, and flavonol-related candidate ion features showed side-associated response patterns (**Figure 1**). The monolignol/phenylpropanoid-related candidate ion tentatively assigned to caffeyl alcohol and the phenolic-acid-related candidate ions tentatively assigned to 4-hydroxycinnamic acid and dihydroferulic acid showed higher normalized relative responses on the yellow-colored side, whereas the flavonol-related candidate ion tentatively assigned to myricetin-3-O-(4’’-acetyl)rhamnoside showed a higher response on the red-colored side.

**Figure 6:**
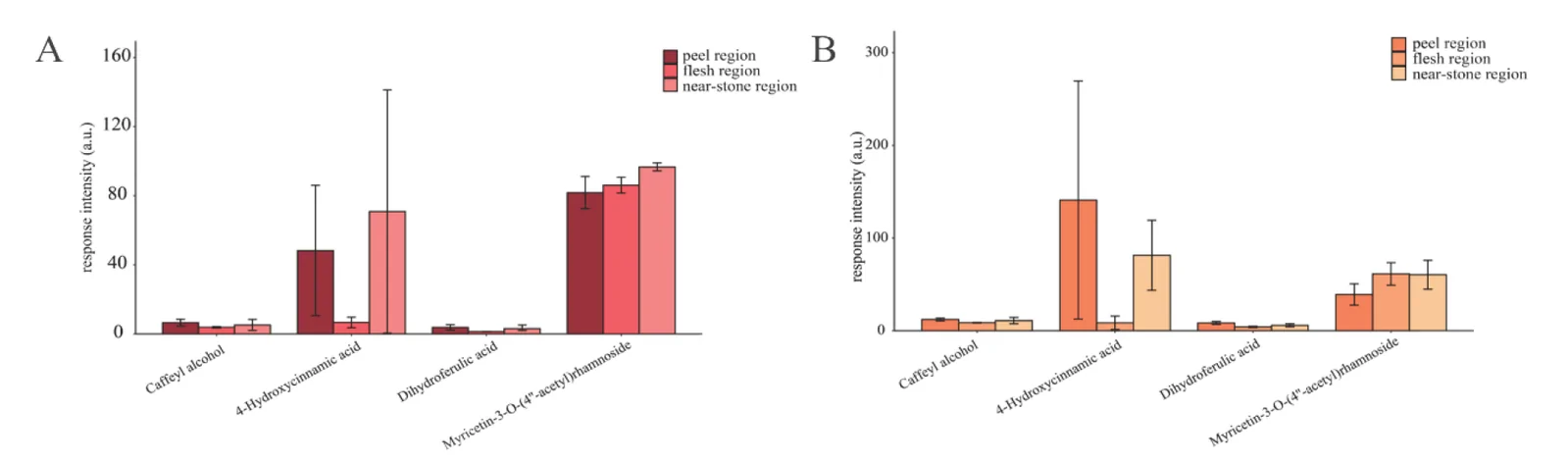
Regional responses of selected monolignol/phenylpropanoid-, phenolic-acid-, and flavonol-related candidate ion features on the red- and yellow-colored sides of apricot fruit. **(A)**, RMS-normalized relative responses of the selected candidate ion features in the peel, flesh, and near-stone regions on the red-colored side; **(B)**, corresponding responses in the three anatomical regions on the yellow-colored side. The selected candidate features were tentatively assigned to caffeyl alcohol, 4-hydroxycinnamic acid, dihydroferulic acid, and myricetin-3-O-(4’’-acetyl)rhamnoside. Data are presented as mean ± standard error from three biological replicates (n = 3). Regional differences are described on the basis of observed mean-response patterns and are not interpreted as statistically validated regional selectivity. Compound assignments are tentative unless confirmed using authentic standards.

Across anatomical regions, the examined candidate ion features showed feature-specific patterns rather than a uniform regional trend (**Figure 6**). For the 4-hydroxycinnamic-acid-related ion, mean responses on the yellow-colored side were 140.91, 8.55, and 81.36 in the peel, flesh, and near-stone regions, respectively. The corresponding values on the red-colored side were 48.28, 6.65, and 70.87. Thus, the 4-hydroxycinnamic-acid-related ion showed the same directional mean pattern on both fruit sides, with lower responses in the flesh than in the peel and near-stone regions. The caffeyl-alcohol- and dihydroferulic-acid-related candidate ions did not show the same consistent regional ordering across both fruit sides.

The myricetin-3-O-(4’’-acetyl)rhamnoside-related ion showed a different regional response pattern. On the red-colored side, its mean responses were 81.80, 86.07, and 96.64 in the peel, flesh, and near-stone regions, respectively, whereas the corresponding values on the yellow-colored side were 39.07, 61.31, and 60.39.

### 3.4 Side- and tissue-associated patterns of candidate sugar-phosphate- and nucleotide-sugar-related ion features

**Figure 7:**
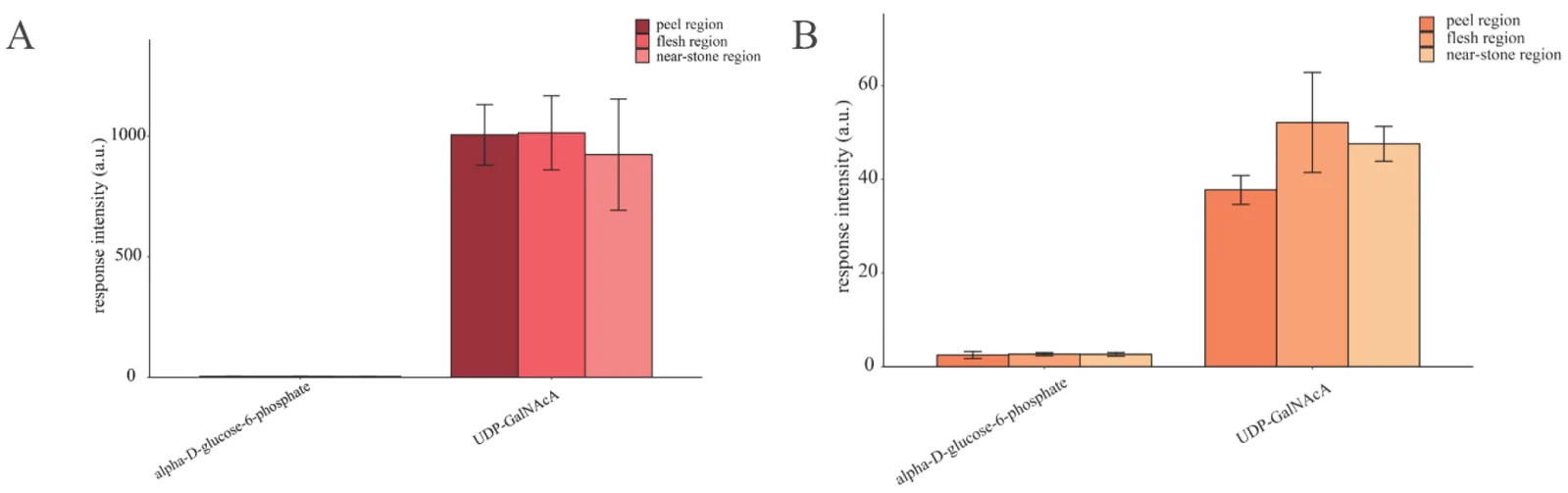
Regional responses of candidate sugar-phosphate- and nucleotide-sugar/glycosylation-related ion features on the red- and yellow-colored sides of apricot fruit. **(A)**, RMS-normalized relative responses of the G-6-P-related and UDP-GalNAcA-related candidate ion features in the peel, flesh, and near-stone regions on the red-colored side; **(B)**, corresponding responses on the yellow-colored side. Data are presented as mean ± standard error from three biological replicates (n = 3). At the current sample size, no statistically significant differences were detected among the three anatomical regions; the data are therefore presented as descriptive regional mean-response patterns rather than evidence of regional selectivity. Compound assignments are tentative unless confirmed using authentic standards.

Two candidate ion features tentatively assigned to alpha-D-glucose-6-phosphate (G-6-P) and UDP-GalNAcA were examined to characterize their side- and tissue-associated ion-response patterns (**Figures 2**, **7**). The UDP-GalNAcA assignment was based on MS1 precursor-mass matching only and was therefore treated as an MS1-only candidate annotation rather than a confirmed compound identification.

The G-6-P-related ion showed only a small difference between the two fruit sides. Across the peel, flesh, and near-stone regions, its RMS-normalized relative responses ranged from 2.97 to 3.70 on the red-colored side and from 2.48 to 2.68 on the yellow-colored side. Regional variation within each fruit side was limited.

In contrast, the candidate UDP-GalNAcA-related ion showed a pronounced side-associated response pattern. Its mean responses ranged from 923.72 to 1013.84 across the three anatomical regions on the red-colored side, compared with 37.73 to 52.13 on the yellow-colored side. The ion-distribution map similarly showed a broader high-response region on the red-colored side (**Figure 2**). Within each fruit side, regional variation was smaller than the red-versus-yellow difference. At the current sample size (n = 3), the regional comparisons for the G-6-P- and UDP-GalNAcA-related candidate ion features did not provide evidence of statistically significant differences among the peel, flesh, and near-stone regions. **Figure 7** is therefore interpreted as a descriptive comparison of regional mean responses rather than evidence of regional selectivity. For the UDP-GalNAcA-related candidate ion, the main pattern was the difference between the red- and yellow-colored sides rather than a consistent anatomical-region gradient.

### 3.5 Side- and tissue-associated response patterns of selected membrane-lipid-related candidate ion features and an unassigned ion feature

Selected membrane-lipid-related candidate ion features, together with one unassigned ion feature retained after annotation quality control, showed distinct side-associated response patterns (**Figure 3**). Most candidate ions with retained compound-level annotations showed higher normalized relative responses on the yellow-colored side than on the red-colored side. The mean responses on the yellow- and red-colored sides were 11.09 and 2.29 for the 5Z, 8Z, 11Z, 14Z-eicosatetraenedioic-acid-related ion, 32.04 and 12.50 for the nervonic-acid-related ion, 3.30 and 1.81 for the LysoPC 16:2(2n isomer)-related ion, and 1.53 and 0.62 for the 1-O-octadecyl-sn-glycero-3-phosphocholine-related ion, respectively. The ion feature at m/z 271.9848 was retained as an unassigned feature after annotation quality control and was therefore not interpreted at the compound level.

The corresponding ion-distribution maps were generally consistent with the relative-response data and showed stronger or broader signals on the yellow-colored side for most of these candidate features. The unassigned ion feature at m/z 271.9848 showed only a small difference between the two fruit sides, indicating that the direction and magnitude of the side-associated response were feature dependent.

**Figure 8:**
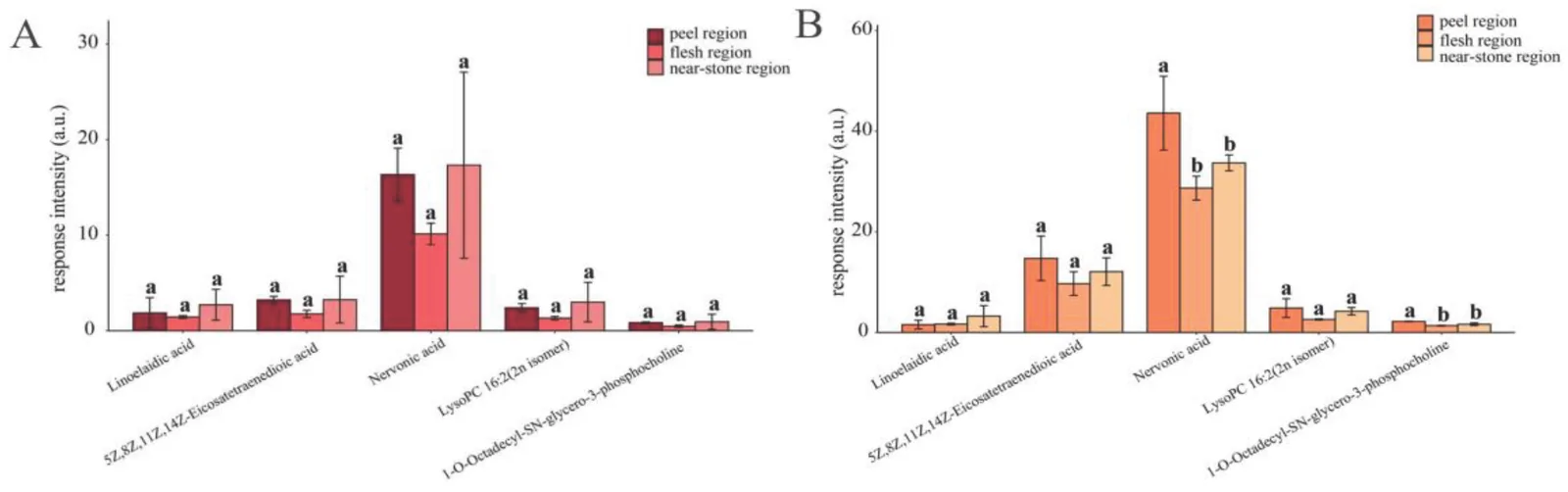
Regional responses of selected membrane-lipid-related candidate ion features and an unassigned ion feature on the red- and yellow-colored sides of apricot fruit. **(A)**, RMS-normalized relative responses of the selected ion features in the peel, flesh, and near-stone regions on the red-colored side; **(B)**, corresponding responses in the three anatomical regions on the yellow-colored side. The selected features comprised the unassigned ion feature at m/z 271.9848 and candidate ions tentatively assigned to 5Z, 8Z, 11Z, 14Z-eicosatetraenedioic acid, nervonic acid, LysoPC 16:2(2n isomer), and 1-O-octadecyl-sn-glycero-3-phosphocholine. Data are presented as mean ± standard error from three biological replicates (n = 3). At the current sample size, the regional comparisons were not statistically validated and are interpreted descriptively rather than as evidence of regional selectivity. Compound assignments are tentative unless confirmed using authentic standards.

Regional mean patterns were feature dependent (**Figure 8**). On the red-colored side, the mean responses of the nervonic-acid-related ion were 16.34, 10.14, and 17.33 in the peel, flesh, and near-stone regions, respectively, whereas those of the LysoPC 16:2-related ion were 2.41, 1.33, and 3.00. Both candidate ion features showed lower mean responses in the flesh than in the peel and near-stone regions. In contrast, the unassigned ion feature at m/z 271.9848 showed relatively small variation among the three anatomical regions.

## 4 Discussion

The present study showed differences in normalized ion-response profiles between the red- and yellow-colored sides of ‘Yanzhihong’ apricot fruit, whereas anatomical-region patterns were less consistent and were limited to individual candidate ion features. Tissue-resolved metabolic heterogeneity has also been reported in other fruit systems, including apple, jujube, strawberry, and peach, although the metabolites involved and their spatial patterns differ among species and tissues (Horikawa et al., 2019; Lu et al., 2023; Fujiki et al., 2025; Sun et al., 2023). In the present apricot dataset, selected monolignol/phenylpropanoid-related, phenolic-acid-related, flavonol-related, sugar-phosphate-related, nucleotide-sugar-related, and membrane-lipid-related candidate ion features showed color-associated differences, whereas regional mean patterns were feature specific rather than uniform across entire chemical classes. Among the selected monolignol/phenylpropanoid-, phenolic-acid-, and flavonol-related features, the caffeyl-alcohol-related candidate ion and the phenolic-acid-related candidate ions tentatively assigned to 4-hydroxycinnamic acid and dihydroferulic acid showed higher relative responses on the yellow-colored side, whereas the myricetin-3-O-(4’’-acetyl)rhamnoside-related flavonol candidate showed a higher response on the red-colored side. Recent metabolomic and transcriptomic studies have indicated substantial cultivar- and developmental-stage-dependent variation in flavonoid and flavonol metabolism in apricot fruit (Chen et al., 2023; Han et al., 2023). These studies provide a relevant biological context for the color-associated variation observed here. However, because the present analysis measured relative MSI ion responses rather than antioxidant capacity or metabolic flux, the observed patterns indicate spatial differences in phenylpropanoid- and flavonoid-related candidate features rather than direct differences in antioxidant function.

The anatomical-region analysis showed that the 4-hydroxycinnamic-acid-related ion was the only feature within this selected set to show the same directional mean pattern on both fruit sides, with lower mean responses in the flesh than in the peel and near-stone regions. The other selected candidates did not show a consistent regional pattern across both sides. Because only three biological replicates were available per side, these regional mean differences were not statistically validated and should not be interpreted as region-selective accumulation. Spatial metabolomic studies in Rosaceae fruits have demonstrated tissue-associated metabolic domains (Sun et al., 2023; Liu et al., 2026), but the present data provide only exploratory evidence of regional variation for individual candidate ions and do not justify extending their behavior to an entire chemical class.

The two carbohydrate-related candidate ion features showed different spatial behaviors. The G-6-P-related feature varied only slightly between fruit sides and among anatomical regions, whereas the candidate UDP-GalNAcA-related feature showed a substantially stronger response on the red-colored side. In plants, nucleotide sugars constitute activated sugar-donor pools that support glycosylation reactions and the biosynthesis of cell-wall polysaccharides. Their biosynthesis, interconversion, and intracellular transport provide substrates for glycosyltransferase-mediated synthesis of hemicelluloses and other cell-wall or glycoconjugate components (Zhang et al., 2021; Ebert and Orellana, 2025). Plant cell-wall construction and remodeling are highly dynamic processes involving coordinated synthesis and organization of cellulose and matrix polysaccharides (Delmer et al., 2024). Therefore, the pronounced side-associated response of the m/z 601.0378 candidate feature provides a biochemical context for considering variation in nucleotide-sugar/glycosylation-related metabolism and in pathways supplying precursors for cell-wall polysaccharide biosynthesis between the two fruit sides. Within each fruit side, the regional differences shown in **Figure 7** were not statistically significant at the current sample size (n = 3). Thus, the present results support a side-associated difference in the UDP-GalNAcA-related candidate ion response, whereas evidence for anatomical-region-associated variation remains insufficient.

However, the UDP-GalNAcA assignment was based on MS1 precursor-mass matching only. Because UDP-GalNAcA and related nucleotide-sugar isomers can share the same molecular formula and exact mass, the present MALDI-MSI data do not permit unambiguous isomer-level identification. The observed distribution should therefore be regarded as a candidate nucleotide-sugar/glycosylation-related spatial association rather than definitive evidence for UDP-GalNAcA itself or for altered cell-wall biosynthetic flux. Establishing such a relationship would require authentic-standard- or targeted-MS/MS-assisted compound confirmation together with direct measurements of cell-wall composition and relevant nucleotide-sugar or glycosyltransferase-related processes.

Most of the selected membrane-lipid-related candidate ion features showed stronger relative responses on the yellow-colored side, although the magnitude of the difference varied among individual features. Membrane-lipid composition and turnover are known to respond dynamically to temperature and postharvest stress in fruit. Recent postharvest studies have further demonstrated extensive remodeling of phospholipids and fatty-acid composition during chilling or browning responses in peach, banana, and longan fruit, including changes in phosphatidylcholine, phosphatidylethanolamine, phosphatidic acid, phosphatidylinositol, and fatty-acid unsaturation (Song et al., 2023; Li et al., 2023; Shuai et al., 2024). These studies provide a broader physiological context for examining membrane-lipid-related candidate features in the present dataset. However, relative MSI ion responses alone do not measure membrane integrity, lipid peroxidation, phospholipid degradation rates, or the rate of senescence. Therefore, the observed differences are interpreted as spatial variation in selected membrane-lipid-related candidate ion features rather than direct evidence of altered membrane physiological status.

Several aspects of the experimental design and analytical workflow define the scope of the present findings. The study examined one apricot cultivar using three paired biological replicates at a single postharvest sampling point, and no parallel measurements of fruit firmness, weight loss, respiration, membrane integrity, or decay were obtained. Therefore, the spatial ion features observed in this study cannot be used to infer temporal changes during storage or to predict postharvest quality and shelf life. The original MSI acquisition did not include a dedicated pooled-QC tissue section or an external reference standard; analytical stability was instead assessed using replicate correlations, feature-level CVs, and endogenous reference ions. In addition, the OPLS-DA model was derived from a small sample set without an independent validation cohort and was therefore used primarily for exploratory candidate-feature prioritization. Only positive-ion MALDI-MSI was performed, and the metabolite assignments were not confirmed using authentic standards. In addition, the 50 μm section thickness was selected primarily to maintain the integrity of the highly hydrated apricot tissue, but no thickness-gradient comparison using thinner sections of the same apricot samples was conducted. Therefore, the present data establish the practical suitability of this section thickness for the current workflow but do not demonstrate its analytical superiority over thinner sections. These limitations define the present study as an exploratory spatial characterization and indicate the need for independent biological, analytical, and physiological validation.

## 5 Conclusion

Taken together, the paired within-fruit MALDI-MSI analysis indicated that the principal spatial contrast in the sampled ‘Yanzhihong’ apricots occurred between the red- and yellow-colored sides rather than among the peel, flesh, and near-stone regions. Within the current dataset, the caffeyl-alcohol-, 4-hydroxycinnamic-acid-, dihydroferulic-acid-, and most selected membrane-lipid-related candidate ions showed higher relative responses on the yellow-colored side, whereas the myricetin-3-O-(4’’-acetyl)rhamnoside-related ion and the MS1-only UDP-GalNAcA-related candidate ion showed higher responses on the red-colored side. Regional ordering was not uniform; among the selected monolignol/phenylpropanoid-, phenolic-acid-, and flavonol-related features, only the 4-hydroxycinnamic-acid-related ion showed the same lower-flesh pattern on both fruit sides. These results define a focused set of candidate spatial features for validation rather than established postharvest-quality markers. Priority follow-up should confirm these candidate assignments using authentic standards and targeted LC–MS/MS, examine their spatial responses in larger multi-cultivar cohorts across defined storage intervals, and relate these responses to firmness, weight loss, respiration, membrane integrity, and decay. Such targeted validation will determine whether the observed side-associated patterns are reproducible and physiologically informative.

## Data Availability

The raw data supporting the conclusions of this article will be made available by the authors, without undue reservation.
