# Supplementary material for: Spatial metabolomic mapping reveals peel-color- and tissue-region-associated metabolic heterogeneity in postharvest ‘Yanzhihong’ apricot fruit
Source: Front Plant Sci. 2026 Aug 20;17:1906148. doi: 10.3389/fpls.2026.1906148 (PMC13538893; doi:10.3389/fpls.2026.1906148)
Supplement: Supplementary file 1 [file Image1.pdf]

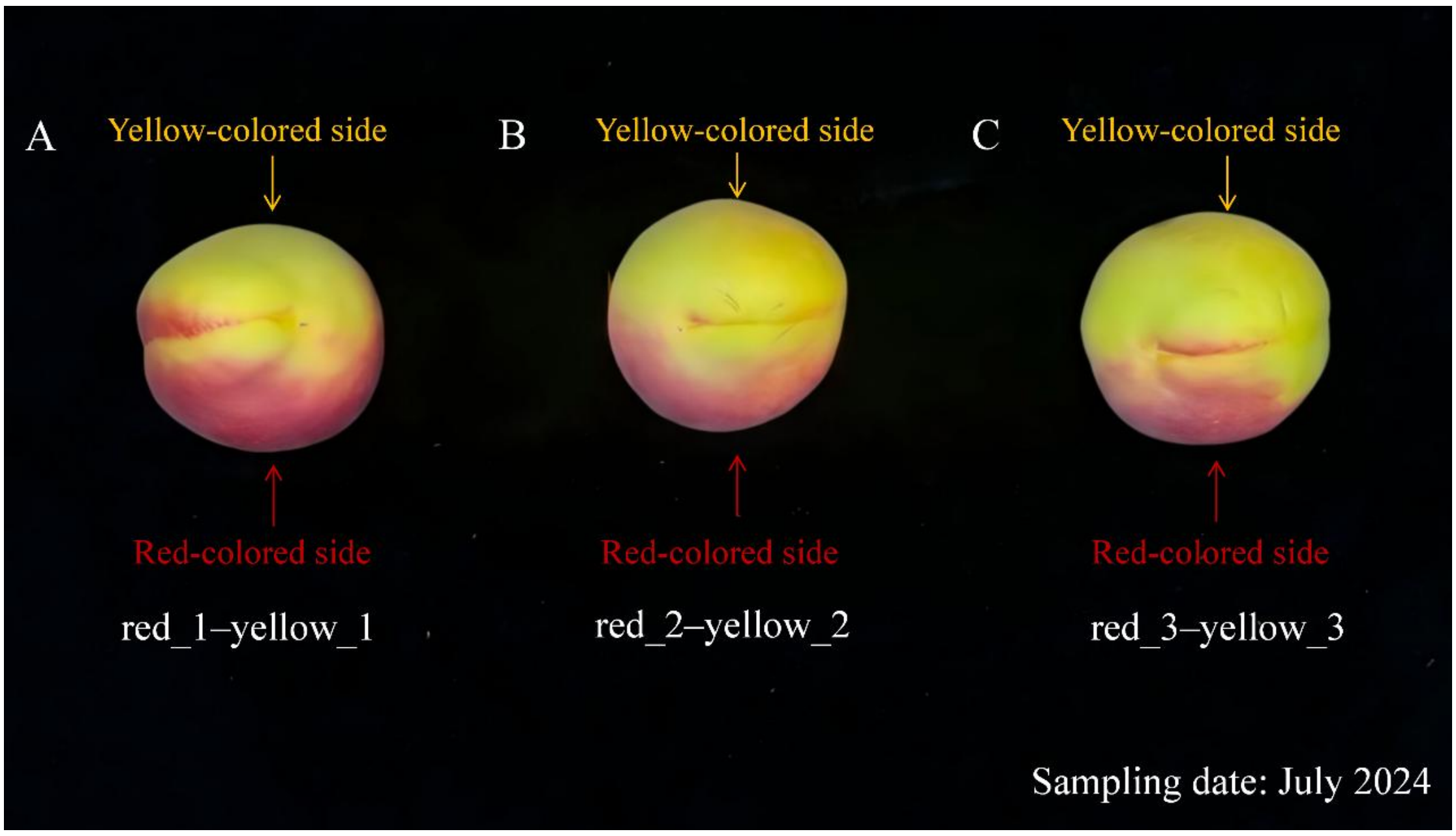

Figure S1. Appearance and paired sampling design of the three biological replicate 'Yanzhihong' apricot fruits used in this study. (A–C) Fruit 1, Fruit 2, and Fruit 3, respectively. The red-colored and yellow-colored sides used for paired sampling are indicated for each fruit. The corresponding paired samples were designated red_1–yellow_1, red_2–yellow_2, and red_3–yellow_3. Fruits were collected in July 2024 and selected according to the same criteria of comparable developmental and maturity status, similar size, intact appearance, and absence of visible disease, insect injury, or mechanical damage.
