# Supplementary material for: Spatial metabolomic mapping reveals peel-color- and tissue-region-associated metabolic heterogeneity in postharvest ‘Yanzhihong’ apricot fruit
Source: Front Plant Sci. 2026 Aug 20;17:1906148. doi: 10.3389/fpls.2026.1906148 (PMC13538893; doi:10.3389/fpls.2026.1906148)
Supplement: Supplementary file 2 [file Image2.pdf]

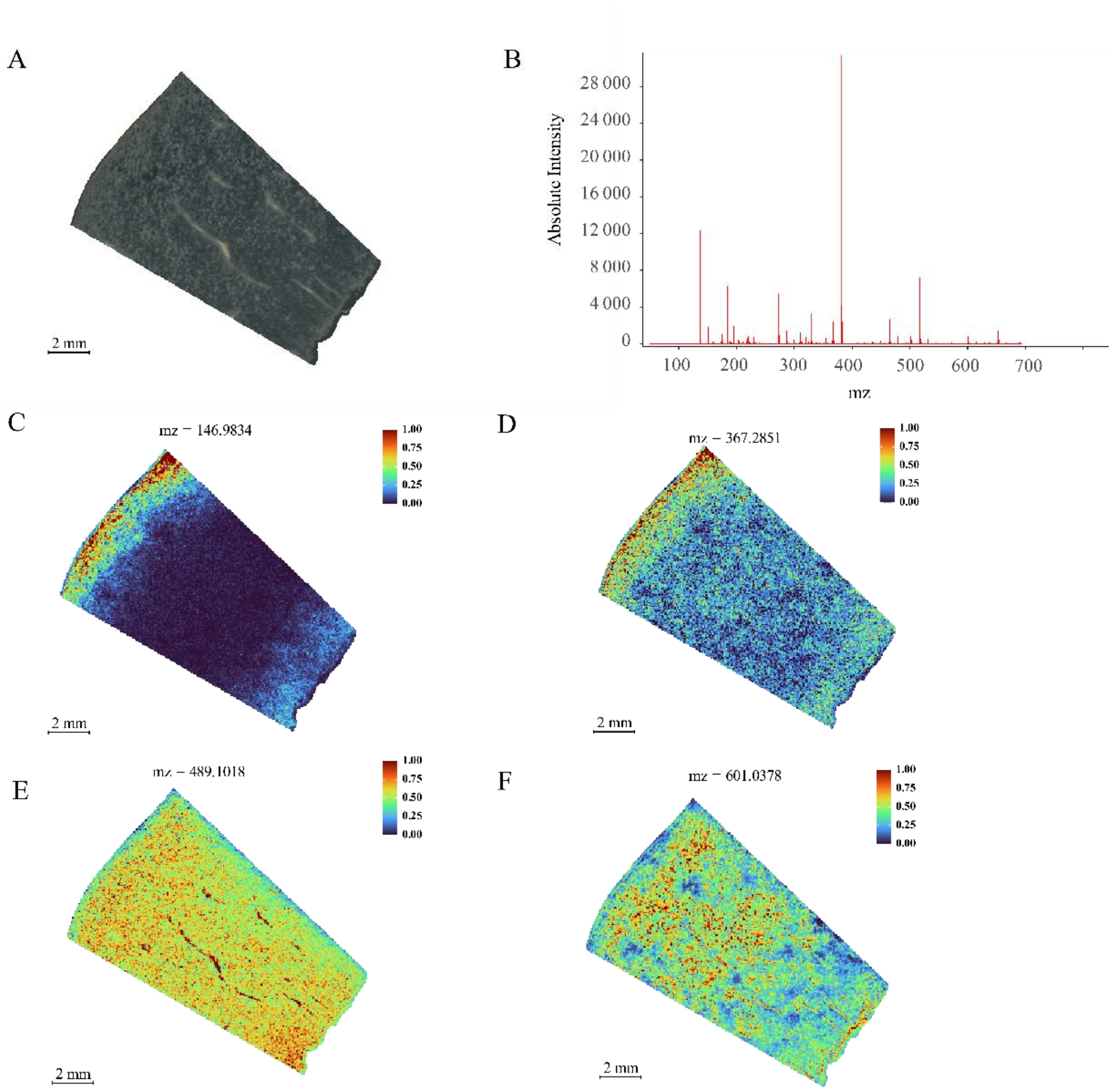

Figure S2. Representative assessment of tissue integrity and MALDI-MSI data obtained from 50 µm cryosections of 'Yanzhihong' apricot fruit. (A) Representative optical image of a 50 µm cryosection mounted on an ITO-coated conductive slide, showing preservation of continuous tissue morphology. (B) Representative mean mass spectrum obtained from the tissue section. (C–F) Representative unsmoothed ion-distribution maps at m/z 146.9834, 367.2851, 489.1018, and 601.0378, respectively. These data demonstrate the practical suitability of the 50 µm sections for the present analytical workflow. Scale bars in panels A and C–F represent 2 mm.
